# Determinants of the practice of exclusive breastfeeding in Guinea: evidence from 2018 Guinean demographic and health survey

**DOI:** 10.1186/s40795-021-00450-2

**Published:** 2021-08-09

**Authors:** Anne Marie Soumah, Mamadou Diouldé Baldé, Mahamadi Tassembedo, Ousmane Ouédraogo, Franck Garanet, Adja Mariam Ouédraogo, Aminata Yara, Mamady Koulibaly, Ibrahima Camara, Seni Kouanda

**Affiliations:** 1 Centre de recherche en santé de la reproduction, Conakry, Guinée; 2 Ministère de la santé, Ouagadougou, Burkina Faso; 3 UNICEF, Ouagadougou, Burkina Faso; 4 Institut Africain de Santé Publique, Ouagadougou, Burkina Faso; 5 Institut de recherche en science de la santé, Ouagadougou, Burkina Faso; 6 Ministère de la sant, Conakry, Guinée

**Keywords:** Exclusive breastfeeding, Determinants, Guinea, Demographic and health survey

## Abstract

**Background:**

Exclusive breastfeeding is critical for infant survival and development. However, the rate of exclusive breastfeeding in the first 6 months of life is low in sub-Saharan Africa. With the current trend in breastfeeding rates in many countries including in Guinea, the World Health Assembly target of at least 50% of children aged less than 6 months being exclusively breastfeed by 2025 is likely to be compromised and lives a numerous infant that are be at risk. The objective of this study was to identify the individual and contextual determinants of the practice of Exclusive Breastfeeding (EBF) in Guinea.

**Method:**

We conducted a secondary analysis of data from the 2018 Guinea Demographic and Health Survey (DHS).

The study population consisted of women who gave birth between the ages of 15 and 49. Our sample consisted of women who had their last birth six (06) months prior to collection. The enumeration areas were our second level. A multilevel logistic regression was performed using Stata version 15.1 software. Three statistical models were implemented: The final model was obtained using the bottom-up step-by-step method. The intra-class correlation coefficient was calculated.

**Results:**

On the 851 women included in our study, 33% reported having exclusively breastfed during the first 6 months of life of their children. After a multivariate analysis, the variables associated with exclusive breastfeeding are: children aged 2–3 months (OR = 0.53 CI95% = [0.36–0.79]) and children aged 4–5 months (OR = 0.23 IC95% = [0.14–0.36]), women in the Faranah area (OR = 2.69 IC95% = [1.21–5.94]) and those in Mamou (OR = 2.27 IC95% = [1.00–5.94]), women who gave birth in a health facility (OR = 1.94 IC95% = [1.34–2.80]) and women living in polygamous households (OR = 0.68 IC95% = [0.48–0.98]).

**Conclusion:**

The practice of exclusive breastfeeding remains low in Guinea. For the achievement of Sustainable Development Goals, particularly the improvement of exclusive breastfeeding practices, the individual and contextual determinants identified in this study should be taken into consideration in policies and programmes.

## Background

Malnutrition is a real public health problem in developing countries. Every year, it is involved in nearly 45% of the 11 million deaths of children under five in these countries [1].. Guinea, like other countries in sub-Saharan Africa, faces a worrying nutritional situation. According to the results of the latest Demographic and Health Survey (DHS) of 2018, 30% of children under five are stunted or chronically malnourished and 13% are severely stunted [2].. Exclusive breastfeeding (EBF) is one of the recommended feeding practices to prevent this situation. The rate of exclusive breastfeeding was 28.3% according to the SMART national nutrition survey [3].. At the current rate of progress in Guinea, the achievement of the targets set by the World Health Assembly of at least 50% by 2025 is likely to be compromised and the lives of children will remain threatened [4].

Exclusive breastfeeding up to 6 months is one of the specific nutrition interventions proven effective in preventing chronic malnutrition according to the Lancet publications [5, 6].. Exclusive breastfeeding reduces child Mortality rates by up to 13% in low income countries [7]. Optimal breastfeeding practices such as early initiation and exclusive breastfeeding are the key and the less expensive interventions for the reduction of infant morbidity and mortality [8, 9].

Breastmilk confers short- and long-term benefits to both child and mother [10], improves the mother-child bond, enhances immunity and reduces the cost of buying artificial milk [11–14].

Globally, only 38% of infants 0–6 months of age are exclusively breastfed [15]. In West and Central Africa 20% of children under the age of 6 months are exclusively breastfed [16]. According to the results of the last two DHS, the practice of exclusive breastfeeding in Guinea remains low compared to other countries in the sub-region [16] and the trend shows a slow evolution. It went from 27% in 2005 to 21% in 2012 and 34% in 2016 [16].

For Guinea, contributing towards achieving the global target of increasing the rate of EBF in the first 6 months up to at least 50% would require achieving a EBF prevalence rate of 44.3% at the national level [17].

To accelerate the achievement of results for children, since 2004 the Guinean government has put in place several interventions to improve the health of children, including infant feeding. These interventions include the implementation of the national breastfeeding policy focused on the protection of exclusive breastfeeding through communication actions on nutrition education, the application of the International Code of Marketing of Breast milk Substitutes (BMS), the promotion of optimum infant and young child feeding practices (IYCF), the Baby-Friendly Hospitals initiative (BFHI) [16, 18]. Despite these efforts, the evolution of breastfeeding practices has been slow and disproportionate in Guinea especially, exclusive breastfeeding.

Hence the need to conduct contextual studies to better understand the determinants of the practice of exclusive breastfeeding in order to guide policies and programs in this area.

Previous studies conducted in several countries such as India, Nigeria and Ethiopia are unanimous on the association between the level of education of the mother, her age, the number of antenatal consultations, the type of assistance to childbirth, socioeconomic status, certain traditional beliefs, practices and rites and the practice of exclusive breastfeeding [18–20].

In Guinea, to our knowledge, no research has been conducted in this context, it therefore seemed necessary to us to conduct the present study in order to fill this scientific gap.

The objective of this study was to identify the individual and contextual determinants of the practice of Exclusive Breastfeeding (EBF) in Guinea using secondary quantitative data.

## Methods

### Type of study

This was a secondary analysis of data from the Guinea Demographic and Health Survey (DHS) conducted in 2018 which is a cross-sectional survey. The collection methodology as well as the reports are available and accessible on the DHS program website (http://dhsprogram.com). For this study, the data from the women’s individual questionnaire were used. For this study, the data from the individual questionnaire of women were used in particular information on the nutritional practices of children, including breastfeeding.

### Study population and sampling

We considered as the study population all women aged 15 to 49 from the individual woman questionnaire. The number of women surveyed was 10,506. The children are attached to their mothers or to those who take care of them in case of death or abandonment by the biological mother. Our analysis focused on women who had their last birth six (6 months) before the collection and who consented to the survey during the collection period.

This study included all mothers of children who had their last birth 6 months before the data collection, and whether or not they practiced exclusive breastfeeding; present during the visit of the investigators and who answered the question on breastfeeding.

**Fig. 1:**
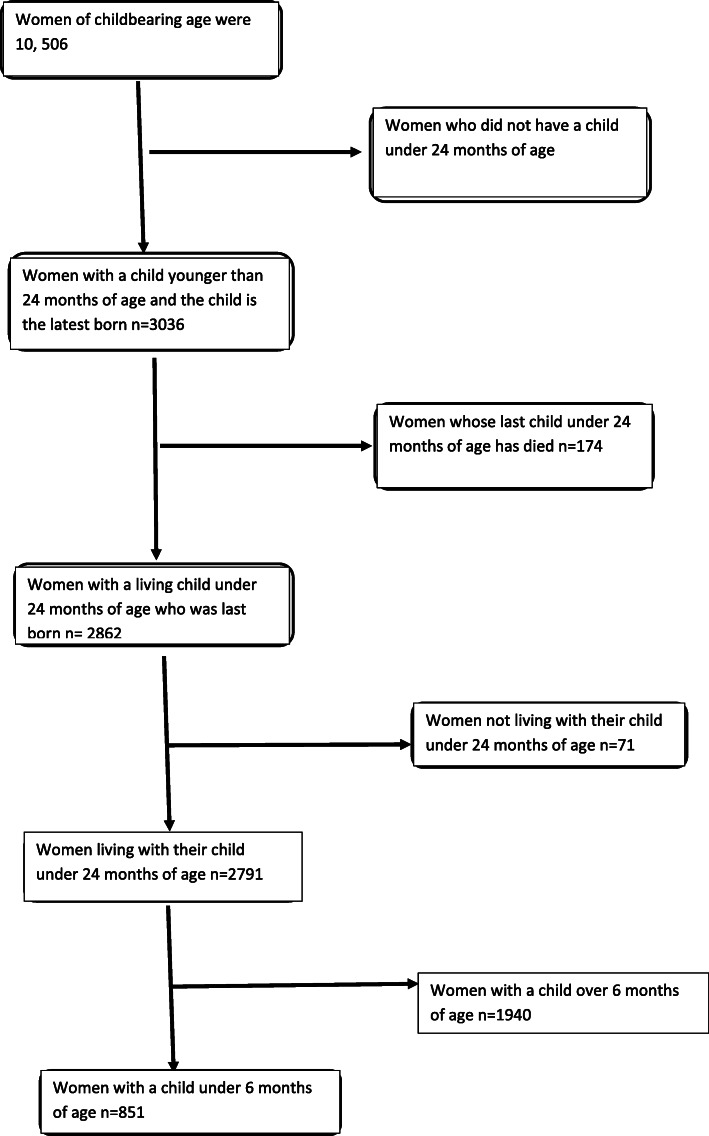
The flow chart for study participants

The following were not included in this study: Mothers whose children were not alive at the time of collection; those whose newborns did not live with them and mothers whose children are over 6 months of age (Fig. 1).

### Study variables

Our dependent variable was exclusive breastfeeding. This variable was obtained from a set of variables related to infant feeding.

First, we created the dichotomous variables that corresponded to the types of food that the mother gave the child the 24 h preceding the survey.

Secondly, we grouped together all the variables whose modality is to have ingested a liquid or a solid other than breast milk. This variable constituted the modality “not having been exclusively breastfed for the variable exclusive breastfeeding. After the construction of the dependent variable, we proceeded to the following recoding:

All mothers who answered that they had not given their child any food other than breast milk were coded 1 and the modality which corresponds to the ingestion of foods other than breast milk for the child was coded 0.

Variables at the individual level (level 1) were the individual characteristics of the mother and child: mother’s age, education level, marital status, parity, region of residence, occupation of woman, type of household (polygamous vs monogamous), number of antenatal consultations, place of delivery, type of delivery assistance, mode of delivery, household size, type of wealth quintile, age and gender of the breastfed child.

Community level variable (level 2) is represented by the enumeration area or community level. The contextual variable in our study is place of residence (urban vs rural).

### Data analysis

For the entire analysis, STATA 15.1 software was used. Before the analysis started, data cleaning, labeling, and recoding were done for all the selected variables. The analysis was carried out in three stages.

The descriptive analysis of all the selected variables was the first step. The socio-demographic and maternal characteristics of the subjects studied were described. The percentages were calculated for each variable.

The univariate analysis consisted in measuring the association between the dependent variable and each of the independent variables selected by performing a simple logistic regression. A variable is retained for the construction of the multilevel model when its *p*-value is less than 20%.

For multivariate analysis, a binary multilevel logistic regression with random effect was performed using the ascending step-by-step method. The adjusted odds ratio (OR) were estimated with their p-value and their confidence interval. Successive models were compared using the likelihood ratio test. This involved multivariate hierarchical analysis to determine the fixed and random effects of the characteristics associated with the practice of exclusive breastfeeding. Using this same analytical technique, we looked for associations between the practice of Exclusive breastfeeding and variables at the individual and community level. Three statistical models have been implemented:

The first model did not contain any independent variables. This allowed us to compare our multi-level regression model to a classic logistic model and to appreciate the variance of exclusive breastfeeding between the different EAs.

The second model was obtained after the introduction of the explanatory variables of the individual level.

The third model or the complete model, which contains in addition to the variables of the individual level, the variable of the contextual level which is the place of residence.

The intra-class correlation coefficient was calculated for each statistical model and the discriminating power of the model was calculated using the Roc table.

### Ethical consideration

This study used secondary data from the DHS, prior to data collection the Health Research Ethics Committee of Guinea provided approval for the implementation of this survey. The database was obtained after a request to the DHS database management program (DHS-Program) which gave us its favorable opinion for the use of this database.

## Results

### The basic characteristics of the sample

A total of 851 women were included in the study. Most mothers with children were aged between 25 and 34 years (44.77%), lived in rural areas (75.79%) and had no formal education (77.91%). Almost all the women (96.83%) were married. More than a quarter of them (27.38%) lived in households considered to be poorer (Table 1).

**Table 1:** Basic characteristics of the sample

Variables	Size (n)	Percentage (%)
**Individual characteristics**		
**Mother**		
**Mother’s age**	(*n* = 851)	
15 to 24 years	301	35.37
25 to 34 years	381	44.70
35 to 49 years	169	19.86
**Mother’s education level**	(n = 851)	
None	663	77.91
Primary	91	10.69
Secondary/Higher	97	11.40
**Marital status**	(*n* = 851)	
Single	27	3.17
Married/Free Union	824	96.83
**Region of residence**	(*n* = 851)	
Boké	114	13.40
Conakry	73	8.58
Faranah	126	14.81
Kankan	144	16.92
Kindia	104	12.22
Labé	101	11.87
Mamou	92	10.81
Nzérékoré	97	11.40
**Parity**	(*n* = 851)	
Primipare	138	16.22
Paucipares	328	38.54
Multipare	385	45.24
**Mother’s work**	(*n* = 851)	
No	327	38.43
Yes	524	61.57
**Type of household**	(*n* = 851)	
Monogame	503	61.49
Polygamous	315	38.51
**Antenatal consultation**	(*n* = 851)	
No ANC	110	13.22
One to three ANCs	444	53.37
Four ANCs and more	297	34.90
**Place of delivery**	(*n* = 851)	
At home	419	49.24
In a health facility	432	50.76
Mode of delivery	(*n* = 849)	
Base track	825	97.17
Caesarean section	24	2.83
**Type of delivery**	(*n* = 851)	
Unique	833	97.88
Twins	18	2.12
**Birth assistance**	(*n* = 851)	
Unassisted childbirth	399	46.89
Assisted childbirth	452	53.11
**Poverty quintile**	(*n* = 851)	
Poorest	233	27.38
Poor	191	22.44
Medium rich	169	19.86
Rich	156	18.33
Richer	102	11.99
**Religion**	(*n* = 851)	
Muslim	776	91.19
Christian	75	8.81
**Ethnic group**	(*n* = 851)	
Soussou	155	18.21
Peulh	340	39.95
Malinké	272	31.96
Kissi	39	4.58
Guerezé	36	4.23
Autres	9	1.06
**Size of household**	(*n* = 851)	
2 to 6 people	343	40.31
More than 6 people	508	59.69
**Age of the child**	(*n* = 851)	
0 to 1 month	273	32.08
2 to 3 months	319	37.49
4 to 5 months	259	30.43
**Gender of the child**	(*n* = 851)	
Male	422	49.59
Female	429	50.41
**Characteristics**		
**Community**		
**Place of residence**	(*n* = 851)	
Urban	206	24.21
Rural	645	75.79

### Prevalence of exclusive breastfeeding in Guinea

The results of this study based on secondary analysis of the DHS database show the prevalence of exclusive breastfeeding was 33% [0.31–0.35] in 2018 among women who had their last birth 6 months before data collection.

### Multilevel analysis

Table 2 presents the result of the multilevel binary logistic regression model in the explanation of the practice of exclusive breastfeeding. After controlling for the other variables, the explanatory variables that were statistically associated with the practice of exclusive breastfeeding at the 5% threshold were the child’s breastfeeding age, region of residence, place of childbirth and type of home.

**Table 2:** Multilevel Analysis

Variable	Empty model		Model 1		Model 2	
	OR	IC	OR	IC	OR	IC
**Breastfeeding age of the child**						
0 to 1 month			1		1	
2 to 3 months			0.53***	[0.36–0.79]	0.53***	[0.36–0.79]
4 to 5 months			0.22***	[0.14–0.36]	0.23***	[0.14–0.36]
**Region**						
Conakry			1		1	
Boké			0.70	[0.29–1.67]	0.72	[0.30–1.62]
Faranah			2.67*	[1.18–6.03]	2.69*	[1.21–5.94]
Kankan			2.03	[0.93–4.44]	1.97	[0.92–4.24]
Kindia			1.36	[0.59–3.11]	1.35	[0.61–3.01]
Labé			1.73	[0.76–3.95]	1.69	[0.75–3.80]
Mamou			2.34*	[1.01–5.40]	2.27*	[1.00–5.94]
Nzérékoré			1.91	[0.76–4.77]	1.88	[0.76–4.61]
**Place of delivery**						
Home			1		1	
Health facility			1.90***	[1.32–2.74]	1.94***	[1.34–2.80]
**Religion**						
Muslim			1		1	
Christian			1.60	[0.74–3.48]	1.60	[0.74–3.46]
**Type of Household**						
Monogame			1			
Polygamous			0.68*	[0.47–0.98]	0.68*	[0.48–0.98]
**Woman currently working**						
No			1			
Yes			1.21	[0.84–1.74]	1.21	[0.84–1.74]
**Random effects**						
**Community characteristic**						
Variance residence2					0.11	
Variance of intercept	1.00		0.57		0.17	
Intra-class correlation (%)	23.3%				0.05%	[0.002–0.69]

Note: * *p* < 0.05; ** *p* < 0.01 *** *p* < 0.001.

The age of breastfeeding was associated with exclusive breastfeeding in both univariate and multilevel analysis. After controlling for the other variables, children in the 2 to 3 month age group were 47% less likely to be exclusively breastfed by their mothers during the first 6 months of their life compared to children in the 0 to 1 month (OR = 0.53 95% CI = [0.36–0.79]) with a statistically significant p<0.001. Children in the 4 to 5 month age group had a 77% reduced chance of being exclusively breastfed by their mothers compared to children in the 0 to 1 month age group (OR = 0.23 95% CI = [0.14–0.36] and *p* = 0.0000).

Compared to women in the Conakry region, women in the Faranah region were 2.69 times more likely to exclusively breastfeed their children during the first six (06) months of life (OR = 2.69 95% CI = [1.21–5.94] and *p* = 0.014). Also, Mamou women were 2.27 times more likely to practice exclusive breastfeeding in the first 6 months of their children’s life (OR = 2.27 95% CI = [1.00–5.94] and *p* = 0.049).

Women who had given birth in a health facility were 94% more likely to practice exclusive breastfeeding compared to those who gave birth at home (OR = 1.94 95% CI = [1.34–2.80] and *p* = 0.0000).

Mothers of children living in polygamous households compared to those living in monogamous households had a 32% reduced chance of practicing exclusive breastfeeding (OR = 0.68 95% CI = [0.48–0.98] and *p* = 0.042).

In the empty model, the enumeration areas (EAs) explained 23% of the variance in the practice of exclusive breastfeeding. After inclusion of individual and contextual variables, this variance was reduced to 0.5%. This suggests that part of the practice of exclusive breastfeeding is explained by contextual variables.

## Discussion

The purpose of this work was to determine the individual and contextual factors of the practice of exclusive breastfeeding in Guinea.

### Prevalence

The practice of exclusive breastfeeding in Guinea remains low compared to that desired by the WHO which is 50% by 2025. This low prevalence of Exclusive breastfeeding in Guinea could be explained on the one hand by the low attendance of health facilities for childbirth which is an opportune time to bring mothers to adhere to this practice. On the other hand by the weak implication of the spouse and the family members to support the breastfeeding woman. Two studies conducted in Mali and the Democratic Republic of Congo have shown that the involvement of spouses and family members has an influence on breastfeeding practices [19, 20]. However, the increase in this rate calls for the setting up of campaigns to promote exclusive breastfeeding towards women, including mothers-in-law as well as sisters-in-law, the husband in order to target all those who have an influence on the infant’s food choice. This would allow mothers to adapt a safe and optimal feeding method for their infants, but also to avoid being influenced by incorrect and erroneous information from family members and also from the community.

This prevalence is lower than those reported in Ghana 52%, Liberia 55% and Togo 58% respectively [21–23].

### Associated factors

In this study, the age of the breastfed child, the place of delivery, the region of residence and the type of household were statistically associated with the practice of exclusive breastfeeding.

This study found that the child’s age was statistically associated with the practice of exclusive breastfeeding. Our results show that the practice of EBF decreased significantly from the age of 2 months to 5 months. This correlation between age and exclusive breastfeeding could be explained by the fact that as the child’s age increases, mothers are more likely to start introducing other foods. Indeed, they perceive that breast milk alone may not be enough to meet the nutritional needs of the child. Several studies reinforce the existing correlation between the practice of EBF by a mother and the age of the child like: Those reported in certain countries of West Africa [24–26]. These results suggest a crucial implication of health professionals, paying more attention to breastfeeding mothers by giving them advice to only breastfeed the child without associating it with anything other than medication. They should also help mothers overcome any barriers to the practice of exclusive breastfeeding through outreach sessions or forum theatres or mass communication activities involving the media.

This study found that the region of residence was significantly associated with the practice of exclusive breastfeeding. Women in the Faranah and Mamou administrative regions were more likely to exclusively breastfeed compared to those living in Conakry. Our results could be explained by the fact that these women do not have easy access to breast milk substitutes given the high cost; this makes them give their breasts to the baby during the first 6 months of life. There is also the support of elders which explains the weight that society exerts on the mother in the practice of breastfeeding. Previous research shows that the practice of breastfeeding was influenced by the social environment, support by family members, close friends and professionals, was crucial for the initiation and maintenance of breastfeeding [23, 26–28].

Another argument is that, they do not fully follow the evolution of new technology, which means that they may not adhere to breast milk substitutes. Unlike the women of Conakry, who are much more exposed to activities in the tertiary sector requiring the separation of mother and child for a long time. With the emergence of new technology and culture shock, the women of Conakry do not want to lose their physical form while breastfeeding. They have access to breast milk substitutes. Our results are contrary to those reported by Anthony et al. or mothers who resided in the Volta region were more likely to practice exclusive breastfeeding than mothers in other regions of Ghana who they attributed to the attachment of women to cultural beliefs [23]..

In addition, the difference in coverage of current interventions and the presence of operational Non-governmental organization (NGO) partners for the promotion of exclusive breastfeeding in the different regions could also explain this difference. For example, there are more NGO implementing partners and IYCF counselling, IYCF outreach and mass media radio in the Mamou region than in the other regions. Exposure to IYCF communication and information activities is higher in Mamou. The difference in social norms in the regions may explain this difference [22, 23].

In this study, women who gave birth in a health facility were more likely to practice exclusive breastfeeding than those who gave birth at home. Place of delivery has been found in several studies to be associated with exclusive breastfeeding [27–29]. This This result found in our study could be explained by the involvement of health professionals in raising the awareness of women on the exclusive breastfeeding component during the various pre and postnatal contacts that make ideal times to encourage mothers to adhere to this practice. It is appropriate that the government put in place other strategies in addition to the involvement of health professionals but also broaden that focused on the community. Our results are similar to those found by Tampah-Naah et al. in Ghana, Nkala et al. in Tanzania, and Bethlihem Adugna et al., in Ethiopia [21, 27, 28].

The study found that women living in polygamous households had a reduced chance of exclusive breastfeeding. These results could be attributed to the fact that in polygamous households, unlike monogamous households, women are more active and have less time for breastfeeding because of daily living activities [21, 27, 28].

The results we have achieved are nationally representative.

Despite these important contributions, the study has some limitations. Indeed, the breastfeeding indicators were collected based on the recall of the 24 h preceding the collection, which does not allow us to be completely certain if the child has been exclusively breastfed for 6 months. However, this is the method recommended by the WHO, which allows us to compare our results with those of other studies. In addition, the type of study used in the DHS, which is a cross-sectional study, allows us to establish an association between exclusive breastfeeding and the different explanatory variables, but cannot establish a causal link. Despite these limitations, the results contribute to a better understanding of the factors associated with exclusive breastfeeding in Guinea. It may also help guide the development of promising strategies for possible improvement of this practice in Guinea.

## Conclusion

The prevalence of exclusive breastfeeding remains low in Guinea with 33% compared to the recommendations of the World Health Organization which is 50% by 2025. This study shows that the exclusive breastfeeding decreases as the children get older. The other main predictive factors of EBF identified in our study were: the region of residence, the place of delivery and the type of household. To increase this prevalence, it would be necessary for the government to take into account the main associated factors in strategies for promoting exclusive breastfeeding.

## Data Availability

The data from the Guinea Demographic and Health Survey (DHS) generated and/or analyzed during the current study are available and accessible on the DHS program website (http://dhsprogram.com).

## Abbreviations

BFHI: Baby-Friendly Hospitals initiative
CI: Confidence interval
DHS: Demographic and Health Survey
EA: Enumeration areas
EBF: Exclusive breastfeeding
IYCF: Infant and young child feeding practices
NGO: Non-governmental organization
OR: Odds ratio
WHO: World Health Organization

## References

[1] Communication UD of. Tracking progress on child and maternal nutrition: a survival and development priority: Unicef; 2009.

[2] Institut National de la Statistique Ministère du Plan et du Développement Economique (2019). Enquête Démographique et de Santé et à Indicateurs Multiplies (EDS V).

[3] Direction Nationale de la Santé Familiale et de la Nutrition, Division Alimentation et Nutrition, Ministère de la santé publique (2015). Enquête nationale nutrition-santé, basée sur la méthodologie SMART.

[4] Organisation Mondiale de la Santé. Plan d’application exhaustif concernant la nutrition chez la mère, le nourrisson et le jeune enfant (2014). Plan d’application exhaustif concernant la nutrition chez la mère, le nourrisson et le jeune enfant 2014.

[5] Scaling Up Nutrition (SUN) (2010). Scaling up nutrition: a framework for action.

[6] Bhutta ZA, Das JK, Rizvi A, Gaffey MF, Walker N, Horton S, Webb P, Lartey A, Black RE, Group TL, Maternal and Child Nutrition Study Group. Evidence-based interventions for improvement of maternal and child nutrition: what can be done and at what cost?. The lancet. 2013;382(9890):452–77. 10.1016/S0140-6736(13)60996-4 23746776

[7] Agho KE, Dibley MJ, Odiase JI, Ogbonmwan SM (2011). Determinants of exclusive breastfeeding in Nigeria. BMC Pregnancy Childbirth.

[8] Mullany LC, Katz J, Li YM, Khatry SK, LeClerq SC, Darmstadt GL (2008). Breastfeeding patterns,time to initiation, and mortality risk among newborns in southern Nepal. J Nutr.

[9] Edmond KM, Kirkwood BR, Amenga-Etego S, Owusu-Agyei S, Hurt LS (2007). Effect of early infant feeding practices on infection-specific neonatal mortality: an investigation of the causal links with observational data from rural Ghana. Am J Clin Nutr.

[10] Turck D, Pédiatrie C de N de la SF de (2005). Allaitement maternel: les bénéfices pour la santé de l’enfant et de sa mère. Arch Pédiatrie.

[11] Gartner LM, Morton J, Lawrence RA, Naylor AJ, O’Hare D, Schanler RJ (2005). Breastfeeding and the use of human milk. Pediatrics..

[12] Chantry AA, Monier I, Marcellin L (2015). Allaitement maternel (partie 1): fréquence, bénéfices et inconvénients, durée optimale et facteurs influençant son initiation et son prolongetion. Recommandations pour la pratique clinique. J Gynécologie Obstétrique Biol Reprod.

[13] Sage L (2014). Connaissances sur l’allaitement maternel des femmes allaitantes avant leur sortie de maternité au CHU Estaing de Clermont-Ferrand.

[14] Victora CG, Horta BL, de Mola CL, Quevedo L, Pinheiro RT, Gigante DP, Gonçalves H, Barros FC (2015). Association between breastfeeding and intelligence, educational attainment, and income at 30 years of age: a prospective birth cohort study from Brazil. Lancet Glob Health.

[15] WHO. The data repository. WHO. World Health Organization; 2016. Available from: http://www.who.int/gho/database/en/ (Accessed 10 March Apr 2016).

[16] Ogbo FA, Eastwood J, Page A, Efe-Aluta O, Anago-Amanze C, Kadiri EA, Ifegwu IK, Woolfenden S, Agho KE (2017). The impact of sociodemographic and health-service factors on breast-feeding in sub-Saharan African countries with high diarrhoea mortality. Public Health Nutr.

[17] WHO (2021). The global targets, tracking tool. World Health Organization.

[18] Young MF, Nguyen P, Kachwaha S, Tran Mai L, Ghosh S, Agrawal R, Escobar‐Alegria J, Menon P, Avula R. It takes a village: an empirical analysis of how husbands, mothers‐in‐law, health workers, and mothers influence breastfeeding practices in Uttar Pradesh, India. Matern Child Nutr. 2020;16(2):e12892. 10.1111/mcn.12892 PMC7083414 31773869

[19] Sacko K, Maiga B, Konaté D, Diakité FL, Diakité AA, Doumbia S, Traoré F, Doumbia AK, Togo P, Coulibaly O, Konaré H, Diall HG, Dembélé A, Touré A, Traoré M, Cissé ME, Sangaré A, Doumbia A, Sidibé LD, Dicko FT, Togo B, Sylla M (2019). Breastfeeding practice at the Gabriel Touré Teaching Hospital in Bamako, Mali. J Med Health Sci.

[20] Kanteng GA, Lubala TK, Mutombo AM, Mutoke GN, Kasongo AN, Wembonyama SO, Luboya ON. Perception de l'allaitement maternel et de la diversification alimentaire dans une zone urbaine congolaise. Pan Afr Med J. 2014;19. 10.11604/pamj.2014.19.336.5038 PMC4405067 25918576

[21] Kingsley EA, Osita KE, Pramesh RG, Osuagwu LU, Garry JS, Wadad KT, Catharine F, Felix AO (2019). Exclusive breastfeeding rates and associated factors in 13 “economic Community of West African States” (ECOWAS) countries. Nutrients.

[22] Zhang Z, Yu Z (2018). Lijuan Zhang Hongwei wan what factors influence exclusive breastfeeding based on the theory of planned behavior. Midwifery.

[23] Tampah-Naah AM, Kumi-Kyereme A. Determinants of exclusive breastfeeding among mothers in Ghana: a cross-sectional study. Int Breastfeed J. 2013;8(1):1–6. 10.1186/1746-4358-8-13 PMC3852847 24119727

[24] Onah S, Osuorah DIC, Ebenebe J, Ezechukwu C, Ekwochi U, Ndukwu I (2014). Infant feeding practices and maternal socio-demographic factors that influence practice of exclusive breastfeeding among mothers in Nnewi south-East Nigeria: a cross-sectional and analytical study. Int Breastfeed J.

[25] Mogre V, Dery M, Gaa PK (2016). Knowledge, attitudes and determinants of exclusive breastfeeding practice among Ghanaian rural lactating mothers. Int Breastfeed J.

[26] Gusmão AM, Béria JU, Gigante LP, Leal AF, Schermann LB (2013). The prevalence of exclusive breastfeeding and associated factors: a cross-sectional study of teenage mothers between 14 and 16 years of age in the city of Porto Alegre in the state of Rio Grande do Sul, Brazil. Cienc Saude Coletiva.

[27] Tampah-Naah AM, Kumi-Kyereme A (2013). Determinants of exclusive breastfeeding among mothers in Ghana: a cross-sectional study. Int Breastfeed J.

[28] Adugna B, Tadele H, Reta F, Berhan Y (2017). Determinants of exclusive breastfeeding in infants less than six months of age in Hawassa, an urban setting, Ethiopia. Int Breastfeed J.

[29] Alebel A, Tesma C, Temesgen B, Ferede A, Kibret GD (2018). Exclusive breastfeeding practice in Ethiopia and its association with antenatal care and institutional delivery: a systematic review and meta-analysis. Int Breastfeed J.

